# The effect of observing novice and expert performance on acquisition of surgical skills on a robotic platform

**DOI:** 10.1371/journal.pone.0188233

**Published:** 2017-11-15

**Authors:** David J. Harris, Samuel J. Vine, Mark R. Wilson, John S. McGrath, Marie-Eve LeBel, Gavin Buckingham

**Affiliations:** 1 Department of Sport and Health Sciences, University of Exeter, Exeter, United Kingdom; 2 Exeter Surgical Health Services Research Unit, RD&E Hospital, Exeter, United Kingdom; 3 University of Exeter Medical School, Exeter, United Kingdom; 4 Division of Orthopaedic Surgery, University of Western Ontario, London, Canada; Tokai University, JAPAN

## Abstract

**Background:**

Observational learning plays an important role in surgical skills training, following the traditional model of learning from expertise. Recent findings have, however, highlighted the benefit of observing not only expert performance but also error-strewn performance. The aim of this study was to determine which model (novice vs. expert) would lead to the greatest benefits when learning robotically assisted surgical skills.

**Methods:**

120 medical students with no prior experience of robotically-assisted surgery completed a ring-carrying training task on three occasions; baseline, post-intervention and at one-week follow-up. The observation intervention consisted of a video model performing the ring-carrying task, with participants randomly assigned to view an expert model, a novice model, a mixed expert/novice model or no observation (control group). Participants were assessed for task performance and surgical instrument control.

**Results:**

There were significant group differences post-intervention, with expert and novice observation groups outperforming the control group, but there were no clear group differences at a retention test one week later. There was no difference in performance between the expert-observing and error-observing groups.

**Conclusions:**

Similar benefits were found when observing the traditional expert model or the error-strewn model, suggesting that viewing poor performance may be as beneficial as viewing expertise in the early acquisition of robotic surgical skills. Further work is required to understand, then inform, the optimal curriculum design when utilising observational learning in surgical training.

## Introduction

Despite the shifting emphasis within surgical education towards competency-based training, current curricula still depend upon didactic learning, doing under guidance, and repetition [1], with observational learning—the process of passively watching another individual perform a task—maintaining a core role in developing surgical expertise [2]. Whilst the value of observing an expert model is well established in a wide range of contexts [3,4], there is increasing support for the benefits of observing error-strewn performance [5,6]. Given the role of observational learning in effective and economical surgical training [2], this study aimed to determine the style of visual information leading to the most effective early acquisition of surgical skills on a robotic system.

Robotically-assisted devices are now widely used in minimally invasive surgery due to their benefits for patient outcomes and increased levels of surgeon satisfaction [7–9]. There is, however, limited research on how best to instruct trainees during robotic surgical training, when the user must establish a new range of sensorimotor contingencies [10]. Observational learning, the process of observing an actor perform a task prior to physical practice, provides a way of accelerating this type of motor learning [4,11]. Modelling of actions is an effective method for transmitting information, particularly when complex movements are hard to verbalise. While observation is unlikely to lead to optimal learning on its own [3], it is particularly effective as a supplement to physical practice [11]. This benefit is often attributed to the observer developing a model of the coordination patterns required, or a ‘perceptual blueprint’ [12,13], that serves as a standard of reference. This may occur through overlapping neural circuits for action observation and action production [14,15].

The efficacy of observational learning has previously been demonstrated for surgical skill acquisition [16]. For example, in a minimally invasive surgical task, Snyder et al. [17] found simply observing an instance of expert performance to benefit medical students already trained to proficiency on a virtual reality (VR) simulator. Growing evidence, however, suggests that the observed model need not be an expert, and that in fact, viewing errors may contribute to the development of mechanisms for error detection and correction [3,18,19]. For instance, Buckingham et al. [6] found object lifting errors to be reduced in observers who viewed the lifting errors of others. Evidence for the benefit of error observation in a more complex task has also been found in simulated orthopaedic surgery [20]. Following baseline assessment, participants observed either an expert surgeon or an untrained novice perform a simple localization task, where the novice model displayed more erratic instrument control, slower task progression and poorer target visualisation. LeBel et al. found that the two groups improved to a similar degree in the majority of metrics immediately post-observation, but at a one-week follow-up the novice observation group showed significant improvements over their expert-observing counterparts. Given that observing expertise is suggested to promote a model of coordination patterns, and that observing errors may enable models of error detection and correction, the combined benefits of the two approaches could maximise this style of learning. The efficacy of viewing a ‘learning model’, developing from high-error to low-error performance, indicates a mix of performance may aid learning [18]. Indeed, in a sequential timing task Rohbanfard and Proteau [21] found a mixed novice/expert schedule to be most effective for learning [see also [22,23]. Nonetheless, there is only preliminary evidence for the benefits of mixed model observation. Therefore we aim to evaluate the effectiveness of a mixed model in comparison to novice and expert models alone.

In summary, prior research indicates that learning from errors may provide significant benefits for skill acquisition over sole observation of expertise [3,20,24]. In order to examine observational learning in the context of the acquisition of early robotic-surgical skill learning, we examined performance on a daVinci surgical system (Intuitive Surgical Ltd.) before and after observing novice, expert and mixed models. Furthermore, in order to determine the role played by attention in facilitating improvement, participants’ eye movements were recorded during model observation. It is expected, following LeBel et al. [20], that observing errors will be as beneficial as observing expertise immediately post-intervention, but will facilitate improved retention at a one week follow-up.

### Hypotheses

- All observational learning groups will outperform the control group post-intervention and at a retention test.
- Participants viewing the error-strewn model will outperform the expert model at retention.
- The mixed observation model (novice then expert) will outperform either model alone at retention.

## Methods

### Participants

120 undergraduate medical students (48 males) with no previous experience of robotically-assisted surgery were recruited through advertisement during lectures (mean age = 20.2 years, standard deviation = 2.5). A power analysis using G*Power [25] indicated that to find a group difference in line with LeBel et al. (20) of η^2^ = .10 with power = 0.95, a minimum of 104 participants were required. Ethical approval from the University of Exeter research ethics board was obtained prior to data collection. Participants gave written informed consent at the start of testing and were compensated £15.00 for participation.

### Stimuli and equipment

**Fig 1:**
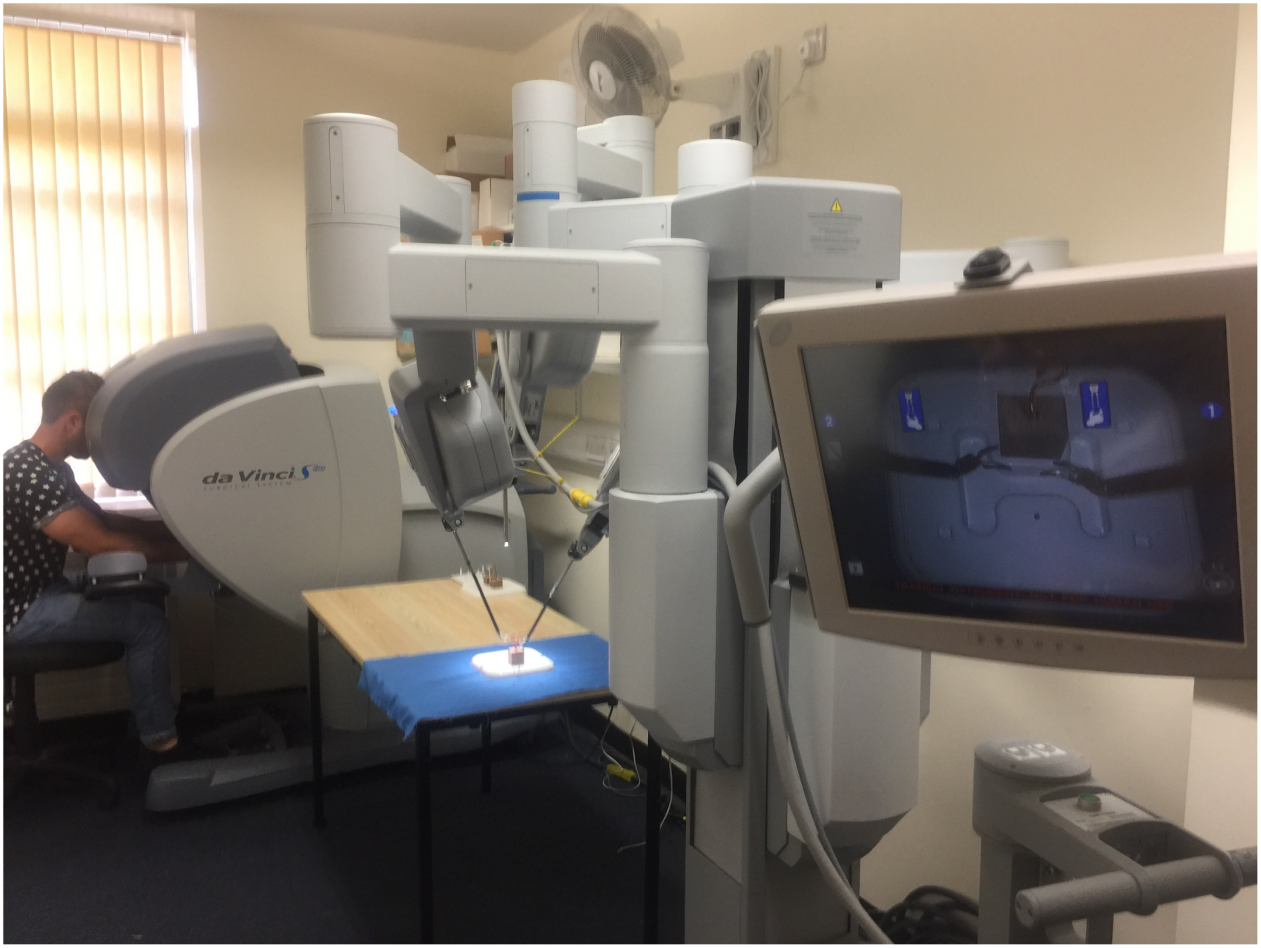
The DaVinci S robotic platform with surgeon console (left) and operating cart (centre).

The experimental tasks were performed on the daVinci S robotically-assisted surgical system (Intuitive Surgical Ltd., Sunnyvale, CA). This system consists of a remote control/viewing console connected to a separate operating cart with three robotic arms (see Fig 1), two of which were connected to a laparoscopic tool (a needle driver with articulated wrist) and one connected to an endoscope. The endoscope records two high definition videos (16:9 aspect ratio) that are played, stereoscopically, in real time to the control console, providing a 3D view of the surgery. The instruments are fingertip controlled with a motion scaling algorithm allowing mapping of hand to instrument movements. The articulated wrist of the laparoscopic instrument allows movements across the same degrees of freedom as the human hand (see [26]).

**Fig 2:**
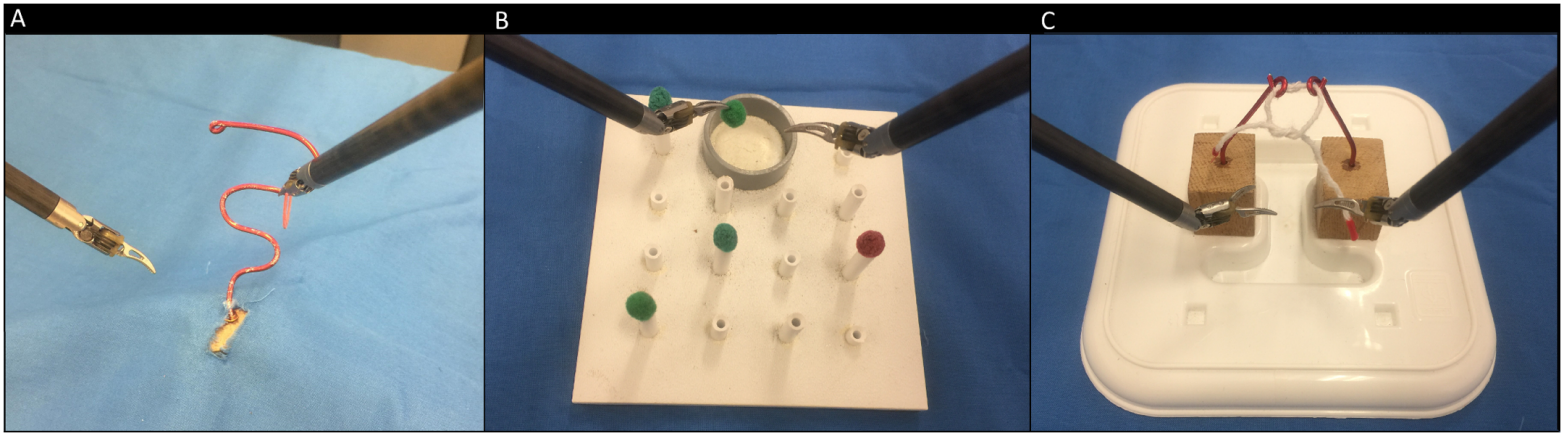
(a) Ring-carrying task (left). (b); Ball pick-up and drop (centre). (c); Knot-tying (right).

We examined performance across one primary task and two separate transfer tasks. The primary task (see Fig 2a) was a ‘ring-carrying’ exercise adapted from the Fundamentals of Robotic Surgery curriculum [27]. A plastic ring is transported along a 15cm curved wire, with the aim of moving the ring from the base to the end of the wire with the right hand, transferring to the left hand and then returning to the starting position without touching the wire. This task is designed to assess proficiency of instrument control. The first transfer task was a ‘ball pick up and drop’ (see Fig 2b), adapted from the Fundamentals of Laparoscopic Surgery curriculum [28] and used previously to assess robotic and laparoscopic skills [9,29]. Five foam balls sat on raised platforms and had to be picked up and transferred to a receptacle. The second transfer task was a ‘knot tying’ exercise (see Fig 2c) adapted from the Fundamentals of Robotic Surgery curriculum, which requires a simple half-knot to be tied in a piece of string suspended from two wires. This task was designed to mimic basic suturing skills.

Video stimuli for the observational learning intervention were developed by recording performance of the primary task (ring-carrying) with a GoPro Hero 3+ camera from novice and expert models. The video view displayed the ring-carrying task and the instruments (as in Fig 2a). The novice video was selected from a number of trials collected from novice performers (non-surgeons with no prior experience) during pilot testing that showed typical errors (e.g. repeated touches of the wire, erratic movements). The expert video featured performance by a consultant urologist, an expert with the daVinci system. Each video showed the task being completed twice. The mixed video showed one instance of the novice video followed by one instance of the expert video.

### Procedure

Participants were required to attend two testing sessions (approximately 30 minutes each) at the Surgical Health Service Research Unit (HeSRU) of the Royal Devon and Exeter Hospital. Visit one consisted of a baseline test on the primary ring-carrying task, followed by the observation intervention and a post-test on the ring-carrying task. Visit two, which occurred seven days after the first visit (as in [20]), assessed retention of learning on the primary task followed by two transfer tasks.

At the start of testing participants were assessed for stereoscopic acuity using the Randot stereoacuity test (Stereo Optical Co, Inc., Chicago, IL). The test measures participants’ ability to discriminate a stereogram circle from two distractors across increasing levels of difficulty (from 400 to 20 seconds of arc), using a 1–10 scoring system. Participants then watched a short introductory video explaining the experimental procedures. Participants were then given one minute to familiarise themselves with the console controls prior to the baseline test (as in [9]). Task performance was recorded with a GoPro Hero 3+ camera set to the side of the stimuli (out of view), for later offline analysis. Instrument movement parameters were recorded with a pair of GENEActiv accelerometers attached to each of the robot arms, which recorded tri-axial accelerations at 100 Hz.

Following the baseline ring-carrying test, participants were randomly assigned to one of the four observation conditions using random number generation. During the three observational learning conditions (novice, expert and mixed) participants watched a video of the ring-carrying task, whilst the control group were given a word search task for the mean duration of the videos. Participants in the video observation groups had point of gaze recorded using SMI ETG 2.0 eye-tracking glasses (SensoMotoric Instruments, Boston MA) that record onto a customized Samsung Galaxy smartphone. The glasses are lightweight (76 g) and record binocular eye movements and the visual scene at 30 Hz to a spatial resolution of 0.5°. During the videos, participants placed their head in a chin rest to minimise head movement and ensure attention was appropriately focused on the content of the videos. All videos were presented on an HD 24 inch Acer monitor placed 48 cm from the participant.

### Data analysis

Performance scores on the three experimental tasks were obtained from video analysis following testing. For the ring-carrying task, time to completion was obtained from the recording, then errors were manually coded (number and type of error). A small touch of the wire scored one point and a drag along the wire or dropping the ring scored two points. The number of errors was divided by the time to completion to create an *errors per second* score. For the knot tying task, time to completion was similarly recorded, with a performance score obtained from a scoring system for surgical knots adapted from Tytherleigh et al. [30,31]. Points were assigned for completing the subsections of the knot correctly, without fumbling or making repeated attempts. For the ball drop transfer task, time to completion was recorded and errors coded using one point for a missed grab of the ball and two points for dropping the ball.

Surgical instrument proficiency was assessed using movement parameters obtained from accelerometry. Tracking of implement movement is widely used for assessing surgical skill, with path length and number of movements particularly linked to expertise [32,33]. Two movement variables were chosen to assess performance in this task. Firstly, jerk, a measure of change in acceleration over time, has previously been linked to surgical performance [34,35] and indicates smooth instrument movements [36]. Secondly, entropy, a measure of randomness/complexity, has been linked to skill learning and automaticity [37] and has previously been used to quantify surgical performance [38]. Accelerometry data was collated and synchronised using GENEActiv PCSoftware, and then analysed using Matlab. Raw data was filtered using a 2^nd^ order, 3Hz Butterworth low pass filter to reduce noise [39], before converting to the Euclidean Norm Minus One [ENMO], which integrates x, y, and z plane accelerations and removes the net effect of gravity [40]. The ENMO provides a measure of total acceleration in any direction. Matlab was also used to calculate jerk [derivative of accelerations with respect to time] from the ENMO, using the formula Jerk = ΔAcc/ΔTime. Sample entropy of accelerations, which is robust to varying sample lengths, was calculated using the natural logarithm of the conditional probability that a series similar for *n* points remains similar at the next point [see [41]].

Allocation of attention has been linked to effective observational learning [42], which can be assessed through point of gaze [43]. Gaze behaviour has previously been used to discriminate attentional strategies of novice and expert surgeons [44] and can indicate which areas of the task observers pay attention to [45]. Gaze data was analysed using SMI BeGaze 3.6 software, which provides automatic fixation detection, and allows user defined areas of interest (AOIs) to be assigned to the visual scene. Dynamic AOIs were assigned to the ring and robot end effectors to determine the proportion of time spent fixating relevant/irrelevant information and the frequency of switches between areas. Data that displayed a poor calibration was removed from the analysis (n = 8).

Statistical analysis was performed using JASP [46]. Outliers (+/- 3 standard deviations from the mean) in performance (n = 5) and accelerometry (n = 3) were removed. Dependent variables were analysed using 3 (trial) x 4 (group) mixed ANOVAs, with one-way ANOVA used to examine interactions and Bonferroni-Holm corrected post-hoc tests where appropriate. Violations of sphericity were corrected for using a Greenhouse-Geisser correction factor. In order to determine equivalence of null effects, Bayes Factors (BF_10_) for main effects and paired comparisons were also obtained using a symmetric Cauchy prior. In addition, to evaluate the extent to which visual attention relates to subsequent learning, Spearman’s correlation coefficients were computed between gaze metrics and performance.

## Results

One way ANOVA indicated there to be no group differences in age (p = .51, BF_10_ = 0.10) or stereovision (p = .40, BF_10_ = 0.14), showing the groups to be well matched (see Table 1).

**Table 1:** Participants’ demographic information.

	Novice (n = 30)	Expert (n = 30)	Mixed (n = 30)	Control (n = 30)
Age†	20.57(2.54)	20.43(3.05)	20.03(2.31)	19.67(2.19)
Stereoscore†	5.30(2.52)	5.37(2.16)	5.20(2.46)	6.10(2.50)
Male/Female†	15/15	11/19	11/19	12/18
Left/Right handers†	3/27	3/27	4/26	3/27

^†^ No difference across groups, all p values >.40

**Fig 3:**
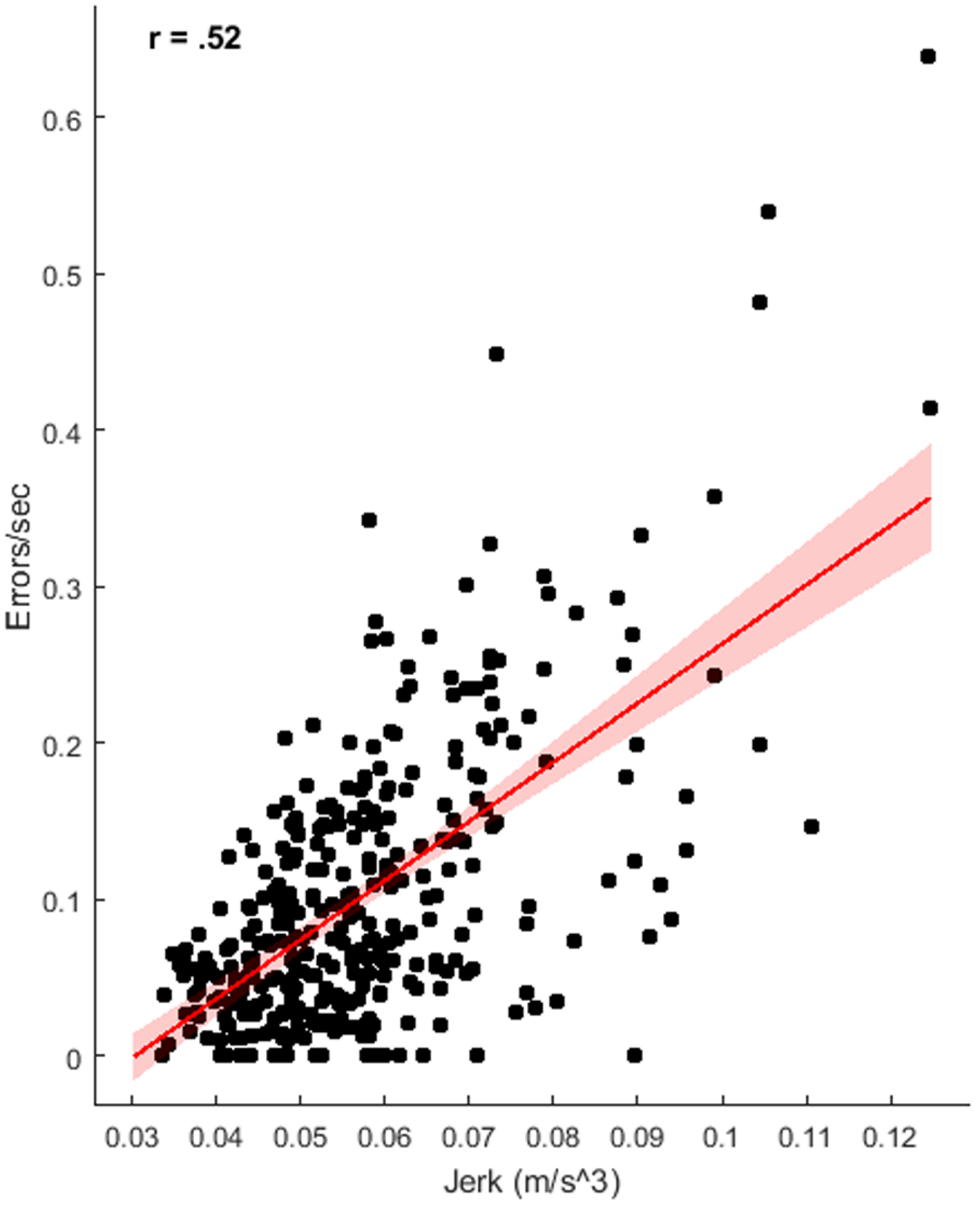
Relationship of instrument jerk with rate of errors, displaying regression line with 95% confidence intervals.

**Fig 4:**
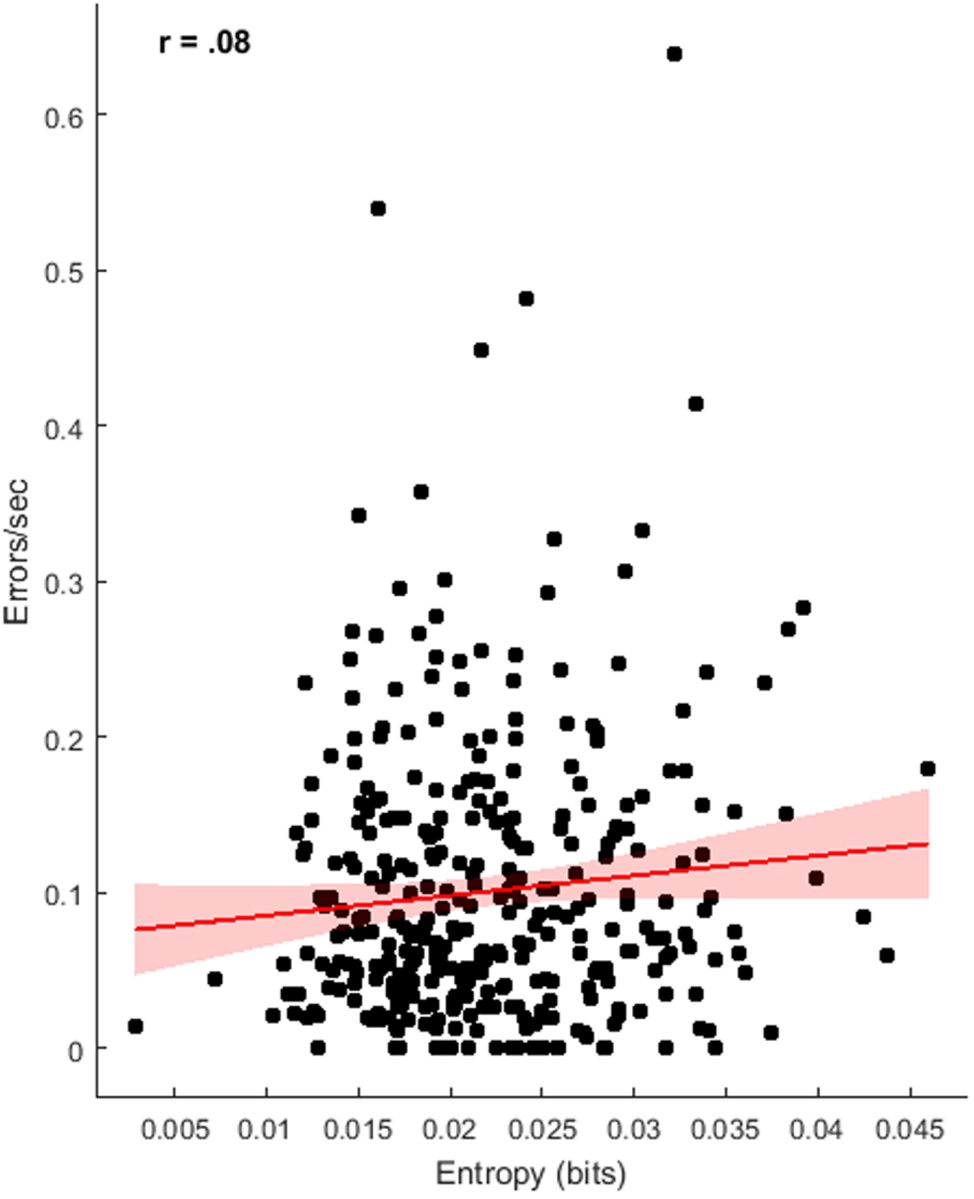
Relationship of instrument movement entropy with rate of errors, displaying regression line with 95% confidence intervals.

To determine the relationship between our objective measures of instrument movement and the subjective coding of error performance, correlational analysis was used across the four groups. There was a moderate relationship between jerk and performance (see Fig 3), indicating increased jerk was related to more errors per second, r(351) = .52 p < .001, BF_10_ = 5.25e+28. There was, however, no clear relationship between acceleration entropy and errors per second, r(351) = .08, p = .11, BF_10_ = .30 (see Fig 4).

**Fig 5:**
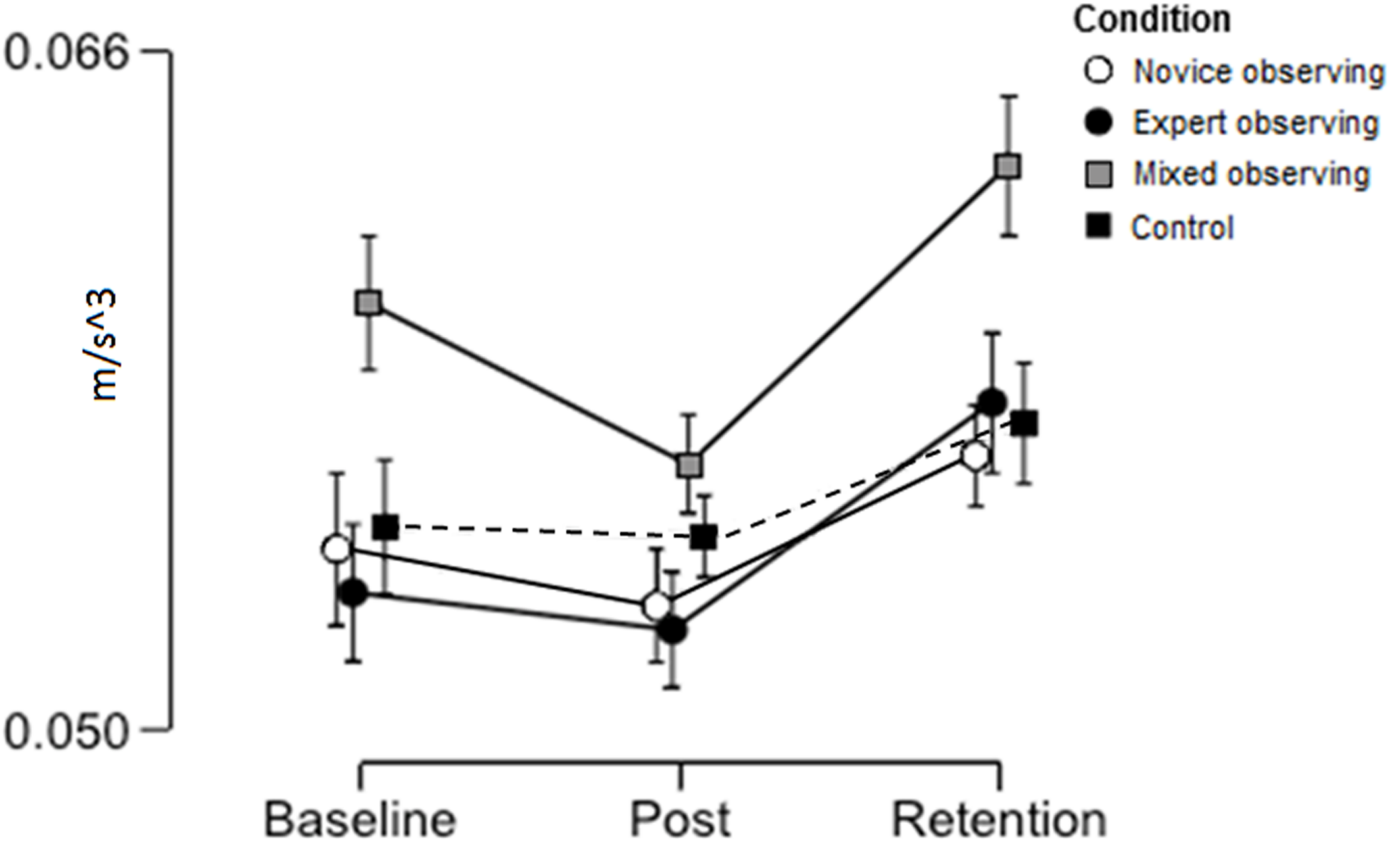
Mean (and standard error) of instrument jerk (m/s^3^) across experimental trials.

To measure group differences in movement parameters a 3 (trial) x 4 (group) mixed ANOVA was conducted on mean jerk (m/s^3^) (see Fig 5). There was a significant main effect of trial, F(2,232) = 5.02, p = .007, ꙍ^2^ = .033, BF_10_ = 2.96, with jerk increasing at retention. There was no main effect of group, F(3,116) = 0.80, p = .50, ꙍ^2^ = .000, BF_10_ = .17 and no interaction, F(6,232) = 0.71, p = .64, ꙍ^2^ = .000, BF_10_ = .03. Post-hoc tests on the effect of trial showed no difference from baseline to post-intervention (p = .12, BF_10_ = 0.23), but there were significant differences from baseline to retention (p = .02, BF_10_ = 2.90), and post-intervention to retention (p < .001, BF_10_ = 24511.24).

**Fig 6:**
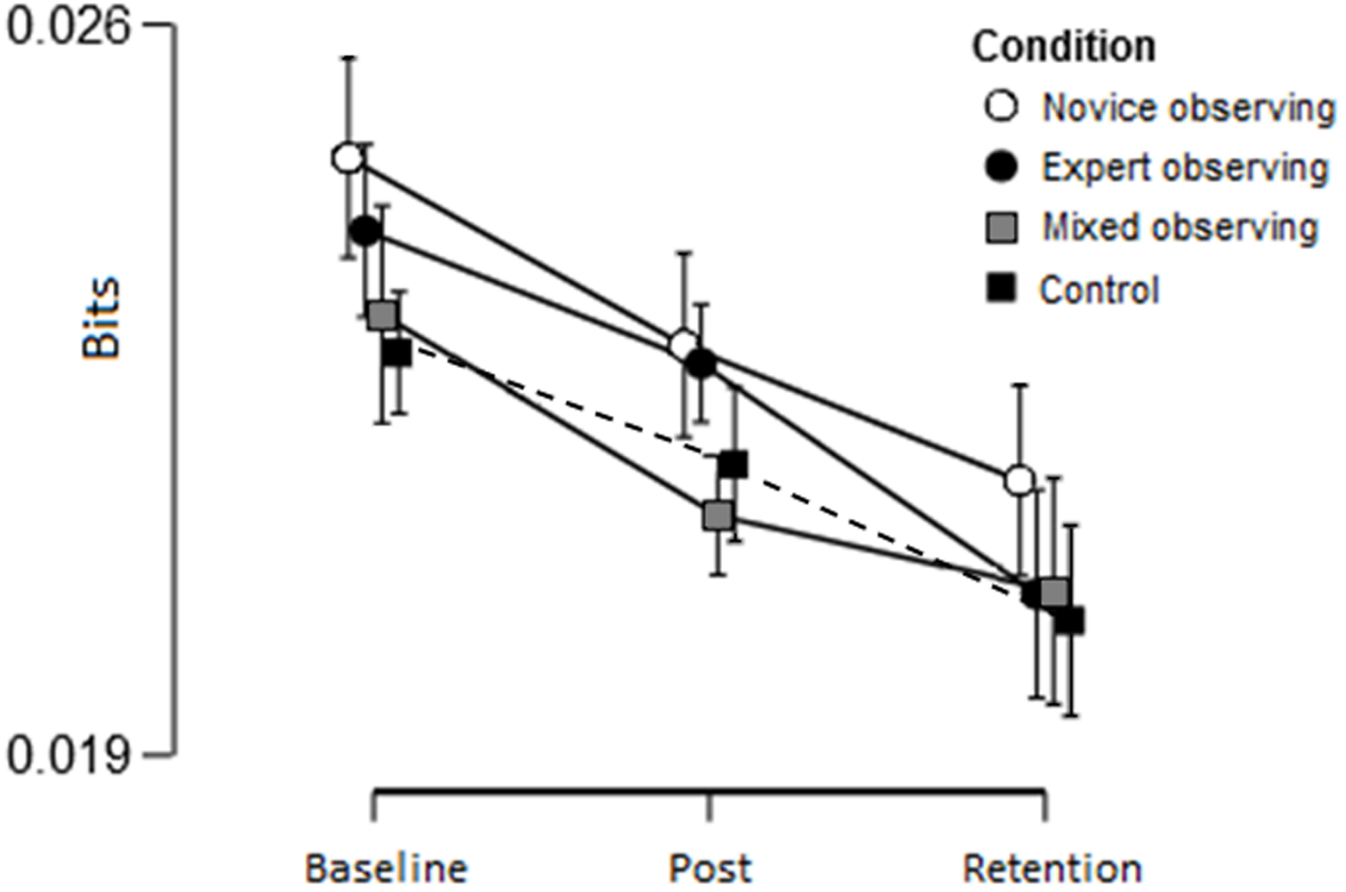
Mean (and standard error) of instrument entropy (bits) across experimental trials.

Similarly, a 3 (trial) x 4 (group) mixed ANOVA was conducted on acceleration entropy (see Fig 6), indicating a significant main effect of trial, F(2,232) = 11.82, p < .001, ꙍ^2^ = .084, BF_10_ = 1443.24, with entropy decreasing over trials. There was no main effect of group F(3,116) = 0.49, p = .69, ꙍ^2^ = .000, BF_10_ = .09, or interaction effects, F(6,232) = 0.16, p = .99, ꙍ^2^ = .000, BF_10_ = .01. Post-hoc tests on the effect of trial showed a significant difference from baseline to post-intervention (p = .009, BF_10_ = 5.33), baseline to retention (p < .001, BF_10_ = 521.30) and post-intervention to retention (p = .009, BF_10_ = 4.69).

**Fig 7:**
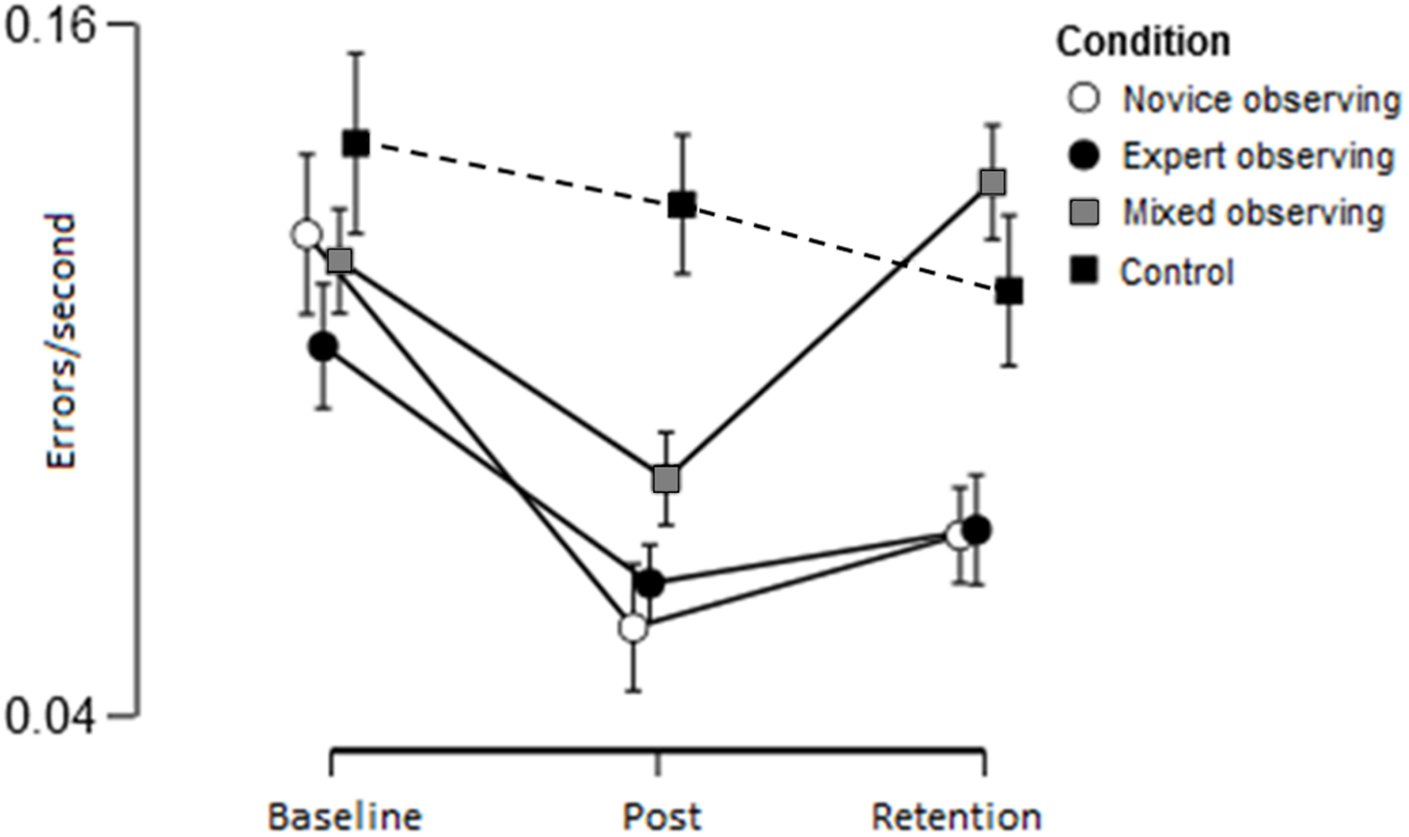
Mean (and standard error) of performance (errors/second) across experimental trials.

To evaluate the effect of the intervention on performance a 3 (trial) x 4 (group) mixed ANOVA was conducted on errors/second scores (see Fig 7). There was a significant main effect of trial F(1.80,194.47) = 13.03, p < .001, ꙍ^2^ = .093, BF_10_ = 1400.70, indicating general improvement from baseline. There was also a significant main effect of condition, F(3,108) = 2.75, p = .05, ꙍ^2^ = .045, BF_10_ = 1.25 and a significant trial by group interaction, F(5.40,194.47) = 2.79, p = .012, ꙍ^2^ = .041, BF_10_ = 2.08.

To examine the condition by trial interaction, follow-up one-way ANOVAs were conducted to test for group differences at each time point. At pre-test there were no significant group differences, F(3,115) = 0.74, p = .53, ꙍ^2^ = .000, with a BF_10_ = .11 providing strong evidence for group equivalence. At post-intervention, there was a significant effect of group, F(3,112) = 3.88, p = .01, ꙍ^2^ = .069, BF_10_ = 3.66, indicating an intervention effect. Post-hoc analysis showed significant differences between Control and Novice (p = .02, BF_10_ = 4.58) and Control and Expert (p = .04, BF_10_ = 2.55) groups, but there were no differences between Novice and Expert (p = .69, BF_10_ = 0.31), Novice and Mixed (p = .49, BF_10_ = 1.34), Expert and Mixed (p = .61, BF_10_ = 0.59) or Mixed and Control (p = .40, BF_10_ = 0.61). This indicates that watching the Novice or Expert video significantly improved performance over the Control condition.

At retention there was also a significant main effect of group, F(3,113) = 3.29, p = .02, ꙍ^2^ = .055, BF_10_ = 1.86. After correction for multiple comparisons, however, post-hoc analysis indicated no significant pairwise differences, although there was a trend towards both novice (p = .07, BF_10_ = 11.44) and expert (p = .07, BF_10_ = 9.00) groups showing a difference from the mixed condition. A Bayes factor of BF_10_ = 0.27 (p = .99) suggests that Expert and Novice groups were equivalent, as were Control and Mixed BF_10_ = 0.30 (p = .99), but there was inconclusive evidence for Control v Expert, (p = .27, BF_10_ = 0.78) and Control v Novice (p = .27, BF_10_ = 0.76).

In the knot tying transfer task, there was no effect of group on time to completion, F(3,116) = 0.18, p = .91, ꙍ^2^ = .000, BF_10_ = 0.06 or knot tying score, F(3,115) = 0.23, p = .88, ꙍ^2^ = .000, BF_10_ = 0.06. For the ball drop task, there was also no effect of group on time to completion, F(3,115) = 1.09, p = .36, ꙍ^2^ = .002, BF_10_ = 0.16 or errors, F(3,113) = 1.48, p = .23, ꙍ^2^ = .012, BF_10_ = 0.24. For both tasks, Bayes factors provide moderate to strong evidence that performance was equivalent across groups.

In order to evaluate the relationship between gaze measures and performance, Spearman’s correlation coefficient was calculated. There was a significant relationship between irrelevant fixation time (away from instruments or ring) and performance at retention, r(79) = .31, p = .006, but not immediately post intervention, r(79) = .18, p = .13. Irrelevant fixations also related to increased entropy in instrument movements post-intervention, r(79) = .35, p = .002. There was no relationship between proportion of time spent observing either the instrument effectors or the ring and subsequent performance (all p values >.30), and no relationship between average fixation duration and performance (all p values >.37).

## Discussion

Surgical skills training has a direct and lasting impact on surgical success and patient outcomes [47,48], but there has been limited investigation of how teaching methods utilised in surgical training curricula can be improved. The acquisition of motor skill in robotic surgery is strongly related to volume of physical practice [49]. Research has shown that observational learning activates the same cortical motor regions as physical practice [50] and allows complex skills to be learnt more quickly [11]. In addition, action observation can contribute to skill learning when physical practice is not possible due to limited training resources, or during online or simulated training programmes. Therefore, this study aimed to build on recent evidence promoting the benefits of observing error-strewn performance [3,6,20] to determine whether viewing errors provided an effective model in a robotically-assisted surgical training task.

Overall, although we observed consistent performance benefits of observation across all of our video-watching groups, the content of what was observed had limited impact. As hypothesised, and in line with previous evidence [19,22,24], observing error-strewn performance provided an immediate learning benefit compared to a non-observation control. This was seen through a reduction in errors per second from baseline to post-intervention. However, unlike Buckingham et al. [6] and Brown et al. [24] there was no benefit of observing errors above that of observing expertise. Comparable learning from novice and expert models has been found previously [19], and LeBel et al. [20] only found group differences after a one-week interval, indicating novice/expert differences may take time to develop in a complex task.

It was also predicted that a mixed observation schedule would afford the greatest learning benefit, as it would provide the ‘perceptual blueprint’ from the expert and the benefits for error detection from the novice. However the results of the mixed group were inconclusive, providing no evidence for the benefit of a mixed schedule over and above the other observation groups, immediately after viewing the videos. Furthermore, there was no difference between the mixed group and the non-observation control groups one week after the intervention, suggesting that observation of a combination of expert and novice performance might impair retention of skills compared to viewing either model in isolation. This finding is in contrast to those of Rohbanfard and Proteau [21] who found a mixed expert/novice model to be more beneficial than observing either model alone. This difference may be partly explained by the very simple sequential timing task used by Rohbanfard and Proteau [21], as findings from simple tasks may not relate well to the acquisition of complex sensorimotor skills [11]. The benefit of a mixed model has also been demonstrated by Domuraki et al. [5] in a central line insertion task. Domuracki et al. [5] found a performance benefit for a mixed model over the expert only model, but only when it was accompanied by specific feedback relating to observed errors. The authors concluded that errors are of benefit when observers know them to be errors, although the results could also be a more general effect of receiving additional feedback. In essence, the present findings may diverge from previous studies due to these task and feedback differences, although the inconclusive benefit of the mixed observation does suggest that there was no additive effect from seeing both novice and expert performance.

Additionally, the current findings differ from the results of LeBel et al. [20] in terms of performance of the task at a one-week follow up retention test. While LeBel et al. found group differences at retention, our results were less clear showing no obvious difference in terms of performance between the novice and expert groups. This apparent similarity between the groups was also evident in terms of performance in either transfer task. As no observation condition outperformed controls at transfer, benefits for learning were likely related to specifics of the ring-carrying task rather than a more general acquisition of skill on the daVinci system.

Our findings may diverge from similar previous studies due to additional factors influencing the mapping of observation to action [15]. For one, unlike the present study, participants in LeBel et al. (20) observed the operator’s hand movements. Hence they saw not only the movement effects [the surgical instruments] but also the effector [the hands of the operator]. Mattar and Gribble [18] suggest that in learning from errors, observers model not only *what* movements to make, but *how* to make them. Therefore the benefit of error observation may have been greater in LeBel et al. as observers not only received information of *what* errors to avoid, but *how* to avoid them. This is supported by unpublished data from LeBlanc et al. [51], who recorded gaze behaviour whilst untrained participants observed the novice and expert video models used by LeBel et al. LeBlanc and colleagues found that time spent observing the model’s hands was related to reduced task completion time and efficient movement in the subsequent simulated arthroscopic task. This effect was driven by the novice observation group, who showed strong relationships between observing the hands and probe distance, time to completion and a global performance score. These relationships did not hold in the expert-observing group, suggesting that when learning from errors, how the error is made provides crucial information.

Another difference between the current study and previous work might relate to the feedback available to the operator. In the surgical simulator used by LeBel et al. [20], participants received haptic feedback of contact with anatomical structures provided by robotic force-feedback devices. In the current study, by contrast, participants received no haptic feedback from the end-effector. While touch feedback is crucial for completion of many basic manual tasks [52], the evidence for the benefits of haptics in robotic surgical performance is not unanimous [53]. A meta-analysis by Van der Meijden and Schijven [54] shows mixed results for the importance of haptic feedback in surgical performance, but a generally positive effect for early phase skill acquisition. Mattar and Gribble [18] describe how simulation is required when adapting information about movement kinematics from visual information, which is likely supported by haptic information given the benefit of haptic feedback for motor skill learning [55,56]. As such, viewing the hands and receiving haptic feedback may have influenced the way errors were simulated, through increased knowledge of *how* the errors were made. Future studies may wish to examine whether the effectiveness of learning from errors depends, in part, on receiving information about *how* to avoid them [18,51].

Consistent with previous work showing that attention can modulate effective observational learning [13,42], we found increased time directing gaze to irrelevant areas of the task (i.e., not on the ring or instruments) was found to be related to increased movement entropy immediately post task, and poorer performance at retention. This relationship illustrates how inattention can be detrimental to learning. Interestingly, there was no obvious benefit of viewing the ring versus the instrument end effectors, in contrast to previous work which has demonstrated benefits of attending to the target of the action in surgical skill learning [57,58]. Thus, although paying attention to relevant aspects of the task is crucial [13], here, attending to the instruments or the ring was equally informative.

An additional outcome of this study is the utility of accelerometry measures for assessing performance in a surgical task. Wrist-worn accelerometers have previously been used for the tracking of gait kinematics [39], but had not been examined in a surgical setting. Motion tracking from surgical simulators has been used previously to calculate measures like path length, force exertion and jerk [20,35,59] that have clear relevance for surgical performance. The current findings, however, suggest that wrist worn accelerometers can provide a simple and cost effective alternative for assessing movement parameters and can contribute to objective assessment of surgical skill. Here, and in line with previous research [36], improved performance was related to reduced instrument jerk. It has been found previously that novices differ from experts in force parameters within laparoscopic surgery, exerting greater force with larger accelerations and jerk [35,36,60]. Trejos et al. [35] found force based metrics like jerk and acceleration to be a better predictor of experience level than task completion time. Assessment of jerk has strong face validity, given that large erratic movements can lead to tissue damage and poor patient outcomes [61]. However, the same relationship was not found for acceleration entropy, a measure of randomness, despite a strong reduction in entropy across trials. While participants’ movements may have become less random with experience and growing confidence [37], absolute measures of force, like jerk, may have more direct relevance for surgical performance.

A limitation of this study may be the restricted physical practice undertaken by participants. Participants may have still obtained major benefits from extra practice that meant group differences were more difficult to observe. Additionally Andrieux and Proteau [22] suggest that the benefits of error-observation are maximised through accompanying physical practice. Future research may wish to intersperse multiple retention tests with periods of physical practice to assess whether group differences emerge. Also, given the differences found between the present study and LeBel et al. [20], future studies could usefully assess the benefit of seeing the kinematics of the operator’s hands for understanding *how* to avoid errors. Further work shall aim to incorporate these findings to evaluate how the use of varied models and different styles of information might enhance the efficacy of training curricula.
